# Supplementary material for: Human Lung Cancer (A549) Cell Line Cytotoxicity and Anti-Leishmania major Activity of Carissa macrocarpa Leaves: A Study Supported by UPLC-ESI-MS/MS Metabolites Profiling and Molecular Docking
Source: Pharmaceuticals (Basel). 2022 Dec 14;15(12):1561. doi: 10.3390/ph15121561 (PMC9784246; doi:10.3390/ph15121561)
Supplement: Supplementary file 1 [file pharmaceuticals-15-01561-s001.zip › pharmaceuticals-2031560-supplementary.pdf]

## SUPPLEMENTARY MATERIAL

# Human Lung Cancer (A549) Cell Line Cytotoxicity and Anti-*Leishmania major* Activity of *Carissa macrocarpa* Leaves: A Study Supported by UPLC-ESI-MS/MS Metabolites Profiling and Molecular Docking

**Mohamed A. A. Orabi [1,*], Omaish Salman Alqahtani [1], Bandar A. Alyami [2], Ahmed Abdullah Al Awadh [3], El-Shaymaa Abdel-Sattar [4], Katsuyoshi Matsunami [5], Dalia I. Hamdan [6] and Mohamed E. Abouelela [7]**

[1] Department of Pharmacognosy, College of Pharmacy, Najran University 1988, Najran 66454, Saudi Arabia
[2] Department of Pharmaceutical Chemistry, College of Pharmacy, Najran University 1988, Najran 66454, Saudi Arabia
[3] Department of Clinical Laboratory Sciences, Faculty of Applied Medical Sciences, Najran University, 1988, Najran 66454, Saudi Arabia
[4] Department of Microbiology and Immunology, Faculty of Pharmacy, South Valley University, Qena 83523, Egypt
[5] Department of Pharmacognosy, Graduate School of Biomedical and Health Sciences, Hiroshima University, 1-2-3 Kasumi, Minami-Ku, Hiroshima 734-8553, Japan
[6] Department of Pharmacognosy and Natural Products, Faculty of Pharmacy, Menoufia University, Shibin Elkom 32511, Egypt
[7] Department of Pharmacognosy, Faculty of Pharmacy, Al-Azhar University, Assiut-Branch, Assiut 71524, Egypt

* Correspondence: maorabi@nu.edu.sa or mohamedorabi@azhar.edu.eg; Tel.: +966-557-398-835

**List of contents**

| No | Content | Page |
|---|---|---|
| Identification of the different metabolites of *C. macrocarpa*. | | S1-4 |
| **Figure S1** | TIC of *Carissa macrocarpa* leaves total hydro-methanolic extract (**T**) and its ethyl acetate fraction (**E**) using UPLC-ESI-MS/MS in negative ionization mode. | S5 |
| **Figure S2** | TIC of *Carissa macrocarpa* leaves butanol (**B**), and methanol (**M**) fractions, using UPLC-ESI-MS/MS in negative ionization mode. | S5 |
| **Figure S3** | Structures of identified compounds and their isomers evaluated by molecular docking simulation. | S6 |
| **Figure S4** | Continue structures of identified compounds and their isomers evaluated by molecular docking simulation. | S7 |
| **Figure S5** | Continue structures of identified compounds and their isomers evaluated by molecular docking simulation. | S8 |
| **Figure S6** | Dose-response curves of total extract and fractions of *C. macrocarpa* against A549 cell line. | S9 |
| **Figure S7** | Dose-response curves of EtOAc and butanol fractions of *C. macrocarpa* against *L. major* promastigote. | S9 |

**Identification of the different metabolites of *C. macrocarpa***

*Phenolic acids/derivatives*

Compounds **6**, **7**, **9**, **39**, **64**, and **74** that showed a molecular ion at *m/z* 353 [M−H]⁻ were detected at retention times 1.87, 2.03, 2.16, 15.06, and 16.52, respectively. Compounds **6** and **9** produced $MS^2$ fragments at *m/z* 191 [quinic acid−H]⁻, *m/z* 179 [caffeic acid−H]⁻, *m/z* 161 [caffeic acid−$H_2O$−H]⁻, hence, assigned as 3-*O*-caffeoylquinic acid and 5-*O*-caffeoylquinic acid, respectively. Where 4-*O*-caffeoylquinic acid (**7**) showed characteristic $MS^2$ fragments at *m/z* 191, 173 [191−$H_2O$] and 161 [1]. Another caffeoylquinic acid derivative, diacetoxy-5-methoxyphenylacroyl-*O*-*p*-coumaroyl-*O*-caffeoylquinic acid (**39**) was identified with the aid of MS, $MS^2$ data (Table 3, main manuscript), and reported literature [2].
Additionally, 4-*O*-caffeoyl shikimic and 3-*O*-caffeoyl shikimic acids have been proposed for **64** and **74** depending upon $MS^2$ fragments *m/z* 179 [caffeic acid−H]⁻, *m/z* 161 [caffeic acid−$H_2O$−H]⁻, the comparison with the published data [3]. Other hydroxycinnamate derivatives such as 5-*O*-*p*-coumaroylshikimic and 4-*O*-*p*-coumaroylshikimic acids have been suggested for compounds **12** and **15**. $MS^2$ fragmentation exhibited a base peak at *m/z* 163 due to [*p*-coumaric acid−$H_2O$], as well as other characteristic fragments [2]. Moreover, compound **29**, which was detected at retention time 6.58 showed a deprotonated molecular ion at *m/z* 515 and distinctive fragments at *m/z* 353 [M−162]⁻ and 179 that are matched with the reported literature of dicaffeoylquinic acid (**29**). Feruloyl-*O*-sinapoylquinic acid (**37**) was designated for peak detected at *m/z* 573 [M−H]⁻, based on $MS^2$ fragments *m/z* 397 [M−ferulic acid−H]⁻, 223 [sinapic acid−H]⁻, 173 [quinic acid−$H_2O$−H]⁻ as compared with the literature [3]. Besides, compounds, dimethoxycinnamoyl-*O*-caffeoylquinic acid (**54**) and its isomer (**70**) were observed at different retention times 13.30 and 15.66 but exhibited similar deprotonated molecular ions at *m/z* 543 [M−H]⁻ and characteristic fragment ions at *m/z* 353 [M−dimethoxycinnamoyl−$H_2O$]⁻ and 173 that attributed to dimethoxycinnamoyl moiety. The compounds **59**, **60**, and **63** were detected at *m/z* 599 and generated $MS^2$ ions at *m/z* 397 and 223, hence assigned as 3-*O*-caffeoyl-4-*O*-sinapoylquinic acid (**59**) and its isomers 3-*O*-Sinapoyl-4-*O*-caffeoylquinic acid (**60**) and 1-*O*-caffeoyl-3-*O*-sinapoylquinic acid (**63**) upon comparison with the published data [3]. In addition, two dimethoxycinnamoylquinic acids isomers were suggested for compound **75** (*m/z* 383 [M−H]⁺) and compound **78** (*m/z* 381 [M−H]⁻) [3]. The hydroxycinnamate glycosides **8**, **13**, **76**, **93**, and **94**, were detected in the current study at retention times 2,13, 2.87, 16.59, 22.78, and 22.90, respectively. Where, compounds **8** and **76** were suggested to be coumaric acid hexoside, due to their similar molecular ions at *m/z* 325 [M−H]⁻ which generated fragment ions at *m/z* 187, 163, and 145 in accordance with the literature [4]. Also, caffeic acid hexoside was assigned for compound **13** depending upon its molecular ion at *m/z* 343 [M+H]⁺ with distinctive fragments at *m/z* 326, 311, and 285, which agree with the reported data [5]. The protonated molecular ions of caffeoyl cyclohexanediol hexoside (**93**) *m/z* 441 [M+H]⁺ and *p*-coumaroyl cyclohexanediol hexoside (**94**) *m/z* 443 [M+H]⁺ produced fragmentation patterns perfectly matching the published data [6]. Compound **77** with *m/z* 337 [M−H]⁻ was characterized as *p*-coumaroylquinic acid, based on comparing its fragmentation patterns (*m/z* 202, 190, and 163) with that in the literature [1]. Finally, compound **83** was identified as caffeoyl-2-hydroxyethane-1,1,2-tricarboxylic acid according to a comparison (Table 3, main manuscript) with the reported data [2] . Quinic acid (**2**) and galloylquinic acid (**44**), gallic acid monohydrate (**31**), hydroxycinnamic acid derivatives [ferulic acid (**32**), caffeic acid (**34**), and syringic acid (**35**)], together with hydroxybenzoic acid (**30**) and ascorbic acid (**47**), were detected at different retention times as shown in (Table 3, main manuscript).

*Flavonoids/derivatives*

Flavonoids are one of the major polyphenolics detected in the polar butanol and MeOH fractions. Compounds **18** and **19** provided the same deprotonated molecular ion at *m/z* 755 $[M{-}H]^-$ with different $MS^2$ fragments; 593 $[M{-}162$ (hexoside)$]^-$ and 285 $[M{-}308$ (rutinoside)$]^-$ for kaempferol-7-*O*-hexoside-3-*O*-rutinoside (**18**) [1]. Quercetin-7-*O*-deoxyhexoside-3-*O*-deoxyhexosyl-hexoside (**19**) generated fragment ions at *m/z* 609 [M−146 (deoxyhexosyl unit)] and *m/z* 301 $[M{-}146{-}308$ (deoxyhexosyl and rutinoside units)$]^-$ [1]. Other kaempferol derivatives were kaempferol-*O*-deoxyhexoside-*O*-deoxyhexosyl-hexoside isomers, which were designated for compounds **21** and **22**, respectively. Both compounds provided the same molecular ions *m/z* 739 $[M{-}H]^-$ and $MS^2$ fragmentation patterns *m/z* 593 and 285 [1]. The compounds **23** and **24** were tentatively identified as quercetin-*O*-deoxyhexosyl-hexoside isomers based on their molecular ion at *m/z* 609 $[M{-}H]^-$ and $MS^2$ fragment at *m/z* 301 due to loss of rutinoside unit (−308 amu). Also, the other isomer exhibited fragment ion at *m/z* 465 in positive ionization mode due to loss of deoxyhexosyl unit (−146 amu) [1].

The compounds **98–100** are belonging to quercetin derivatives and have been identified as rhamnosyl hexosyl-methyl-quercetin, quercetin-3-*O*-galactoside, and quercetin-3-*O*-glucoside, respectively as depicted from their MS, $MS^2$ data and comparison with literature [7]. Compound **98** showed a molecular ion at 613 $[M{+}H]^+$ and $MS^2$ fragments at *m/z* 595, 521, 491, 449, 327, and 287. While compounds **99** and **100** provided the same molecular ion at *m/z* 465 $[M{+}H]^+$ and $MS^2$ fragments at *m/z* 301, 300, 257, 255, 229, 179, and 151.

Kaempferol derivatives were detected upon characterization of MS and $MS^2$ of compounds **79**, **88**, and **89** that are assigned as kaempferol galloylglucoside, kaempferol deoxyhexosyl-hexoside (**88**), and kaempferol-3-rutinoside (**89**), respectively. Kaempferol galloylglucoside (**79**) displayed molecular ion at *m/z* 601 $[M{+}H]^+$ and $MS^2$ fragments at *m/z* 447, 313, 285, 284, 169, 151, and 125 [8]. The two other compounds (**88** and **89**) exhibited the same molecular ion at *m/z* 593 $[M{-}H]^-$, $MS^2$ fragment at *m/z* 285 [1].

The compounds **26** and **58** are belonging to flavonoid glycosides [2] that were tentatively identified as myricetin pentoside [9] and vitexin [10] based on their molecular ions at *m/z* 449 $[M{-}H]^-$ and 431 $[M{-}H]^-$, respectively. Myricetin pentoside showed characteristic $MS^2$ fragment at 317 $[M{-}132]^-$, while 341 $[M{-}90]^-$ and 311 $[M{-}120]^-$ were the main fragment ions of mono-*C*-8-hexosylated flavones such as vitexin [10].

Finally, flavonoid aglycones such as quercetin (**27**) [2], tetramethoxyflavone (**42**), eriodictyol (**48**) [11], myricetin (**95**), and methyldihydroquercetin (**97**) [12] have been proposed for peaks **27**, **42**, **48**, **95**, and **97**, respectively.

*Flavan-3-ols/derivatives*

*C. macrocarpa* is an important source of falvan-3-ol bioactive metabolites responsible for many pharmacological actions of the plant [1,12]. From these interesting molecules, compound **17** has been identified as (epi) catechin based on a molecular ion at *m/z* 289 $[M{-}H]^-$, $MS^2$ fragmentation *m/z* 245, 205, 203, 187, 179, and 161, and the comparison with the literature [1]. Furthermore, the compounds **10**, **11**, **25**, and **53** are belonging to (epi)catechin derivatives of B-type; thus, (epi)catechin dimer was assigned for compounds **10**, **25**, and **53** based on deprotonated molecular ion at *m/z* 577 $[M{-}H]^-$, $MS^2$ fragmentation *m/z* 425, 289 and comparison with the literature [1]. However, compound **11** showed pseudo-molecular ion at *m/z* 865 $[M{-}H]^-$, and fragmentation patterns at *m/z* 451, 425, 407, and 289 were tentatively identified as (epi)catechin trimer [1]. Compounds **14**, **16**, and **20** provided the same deprotonated molecular ion at *m/z* 451 $[M{-}H]^-$ and different $MS^2$ fragments at *m/z* 408, 393, 351, 337, 301, 273, and 245 for compound **14** that was suggested to be catechin-3-*O*-glucoside. However, epicatechin 3-*O*-glucoside and its isomer were designated for compounds **16** and **20** with $MS^2$ fragments at *m/z* 391, 343, 301, 287, 273, and 247 [13]. The compounds **28** and **33** were detected at the same molecular ion at *m/z* 593 $[M{-}H]^-$ and showed $MS^2$ fragments at *m/z* 557, 467, 441, 425, 407, and 289, hence proposed to be (epi)gallocatechin-(epi)catechin [9].

Also, compounds **45** and **49** that were detected at retention times 11.40 and 12.78, respectively, have been identified as (epi)gallocatechin (**45**) [9] and Epigallocatechin-3-*O*-cinnamate (**49**) [14] depending upon MS and $MS^2$ fragments at *m/z* 305 $[M{-}H]^-$, 261, 219, 221, 179, 165, and 125 (**45**) and *m/z* 437 $[M{+}H]^+$, *m/z* 307 and 181 (**49**), respectively. Additionally, three compounds (**50**, **56**, and **61**) which were detected at different retention times displayed the same $[M{-}H]^-$ at *m/z* 471 and were suggested to be methyl-3-*O*-gallocatechin gallate (**50**) ($MS^2$, *m/z* 441, 407, 313, 303, 287, 269, 257, 243, and 161), methyl-3-*O*-epigallocatechin gallate (**56**) ($MS^2$, *m/z* 441, 407, 303, 288, 257, 243, 201, 169, and 161), and methyl-3-*O*-epigallocatechin gallate isomer (**61**) ($MS^2$, *m/z* 441, 407, 297, 269, 241, 213, 199, 168, and 161), respectively [13]. Compounds **57** that detected at retention time 14.37 exhibited $[M{-}H]^-$ at *m/z* 633 and $MS^2$ fragments at *m/z* 481 and 305 due to the loss of galloyl (−152 amu) and glucuronide moieties (−176 amu), hence has been identified as (epi)gallocatechin-*O*-gallate-*O*-glucuronide (**57**). The compounds **65** and **71** that provided the same molecular ions *m/z* 617 $[M{-}H]^-$ and $MS^2$ fragments at *m/z* (599 $[M{-}H_2O]^-$, 465 $[M{-}152]^-$, 289 $[M{-}152{-}176]^-$) were suggested to be (epi)catechin-*O*-gallate-*O*-glucuronide and its isomer upon comparison with the published data [15]. Furthermore, gallocatechin derivatives were tentatively assigned for compounds **69** and **72** based on the protonated molecular ion at *m/z* 455, and $MS^2$ fragments *m/z* 307, 179, and 137 as reported [16]. Dimethyl (epi)catechin was designated for peak **87** that displayed deprotonated molecular ion at *m/z* 317 and $MS^2$ fragment ions at 299, 289, 273, and 245 [16].

*Fatty acids/derivatives*

Compound **5** was proposed to be 3′-(4″-methoxyphenyl)-3′-oxo-propionyl hexadecanoate as previously reported [10]. Several octadecanoic acid derivatives (**36**, **38**, **40**, **43**, **46**, **55**, **66**, **90**, and **91**) were detected mainly in the ethyl acetate fraction of *C. macrocarpa* at different retention times and different molecular ions with characteristic $MS^2$ fragmentation, which may be due to loss of $H_2O$, COOH, and $H_2O$+COOH leading to $[M{-}18]^-$, $[M{-}45]^-$, and $[M{-}63]^-$, respectively, and successive loss of $CH_2$ (−14 amu) [17] . In addition, linoleic (**86**) and palmitic acids (**92**) were identified based on their molecular ions, and fragmentation patterns (Table 3, main manuscript) referenced to published literature [18].

*Sterols and triterpenes/derivatives*

*β*-Sitosterol (**52**) and stigmasterol (**96**) were identified in the ethyl acetate soluble fraction by their molecular ions *m/z* 411 $[M{-}H]^-$ and *m/z* 413 $[M{+}H]^+$ [19,20]. Furthermore, the current study detected several triterpenoid aglycones at different retention times, such as compounds **62**, **80**, **81**, and **82**. Whereas, hydroxybetulinic acid (**62**) had molecular ion at *m/z* 471 $[M{-}H]^-$ with characteristic $MS^2$ fragments at *m/z* 453, 435, 407, and 389 [21]. Additionally, compounds **80**, **81**, and **82** provided the same molecular ion at *m/z* 455 $[M{-}H]^-$, $MS^2$ fragment ions (*m/z* 439, 419, 411, 410, 407, and 397) and designated as ursolic acid, carissic acid, and oleanolic acid upon comparison with the literature [18,22]. Moreover, 3*β*-hydroxyolean-11-en-28,13*β*-olide (**51**) and methyl oleanolate (**67**) exhibited protonated molecular ions at *m/z* 455 and 471, respectively [18,21]. Other pentacyclic triterpenoids that detected at retention time 15.46 (**68**) and 15.92 (**73**) are 3,27-dihydroxy-12-ursen-28-oic acids (3*β*-form), and 27-(4-Hydroxy-3-methoxycinnamoyl) (*E*-form), and its isomer, where they provided the same deprotonated molecular ion at *m/z* 647 with $MS^2$ fragments at *m/z* 485, 470, 455, and 440 [18].

*Miscellaneous*

Lignans such as carinol (**1**) *m/z* 377 $[M{-}H]^-$ and $MS^2$ at 333, 271, 257, 163, and 119 and carissanol (**84**) *m/z* 375 $[M{-}H]^-$ and $MS^2$ at *m/z* 361, 347, 294, and 123 were assigned for (**1**) and (**84**) depending on a comparison

of their data with that previously reported [10]. Secoiridoid, which is also known as oleuropein aglycone (**41**), exhibited deprotonated molecular ion at *m/z* 377 with $MS^2$ fragments at *m/z* 345 $[M-CH_3OH]^-$, 327 $[M-CH_3OH-H_2O]^-$ [10]. Furthermore, sesquiterpenes such as dehydrocarissone which is known as 11-hydroxy-1,4-eudesmadien-3-one (**3**), cryptomeridiol (**4**), germacrenone (**85**) were identified based on MS, $MS^2$ and referenced to the published data [10,21]. Compound **101** with *m/z* 621 $[M-H]^-$ and distinctive fragment ion at 501 was assigned as 2(R)-26-([(2*E*)-3-(4-hydroxy-3-methoxyphenyl)-1-oxo-2-propen-1-yloxy)-2,3-dihydroxypropylester [23]. Finally, compound **102** that detected at retention time at 31.25 gave a molecular ion at *m/z* 429 $[M-H]^-$ and 431 $[M+H]^+$ with characteristic $MS^2$ fragments at *m/z* 205, 191, 177, 149, 121, hence assigned as $\alpha$-tocopherol [24].

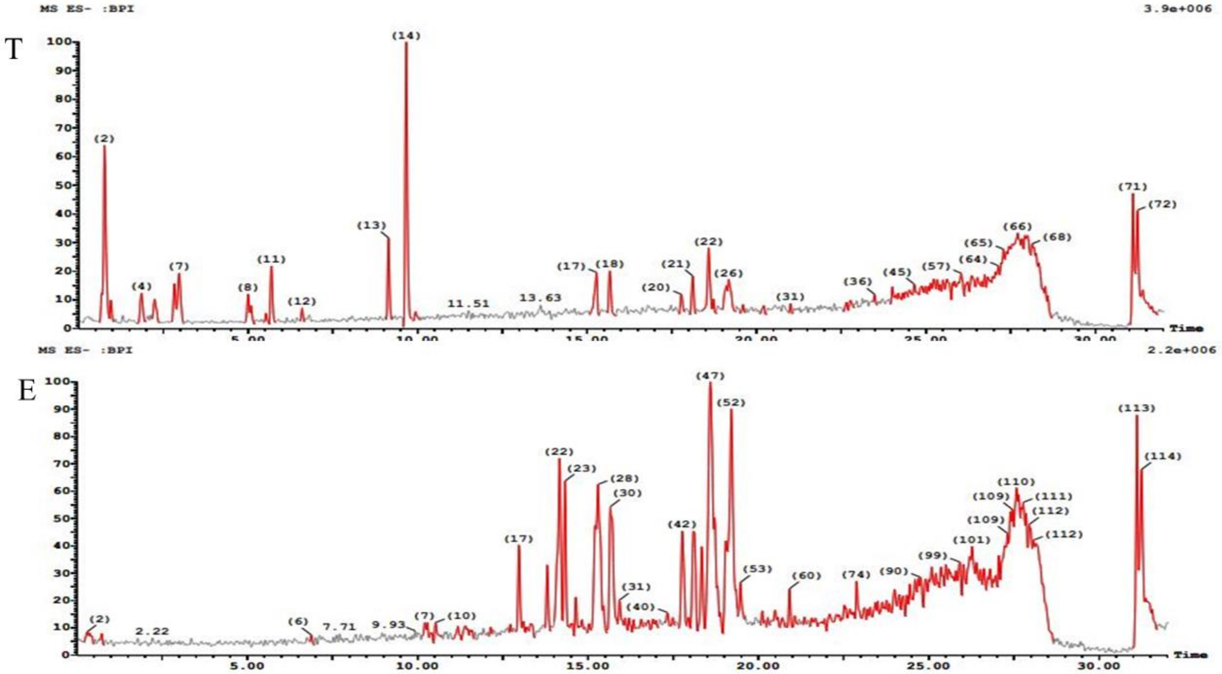


**Figure S1.** TIC of *Carissa macrocarpa* leaves total hydro-methanolic extract (**T**) and its ethyl acetate fraction (**E**) using UPLC-ESI-MS/MS in negative ionization mode.

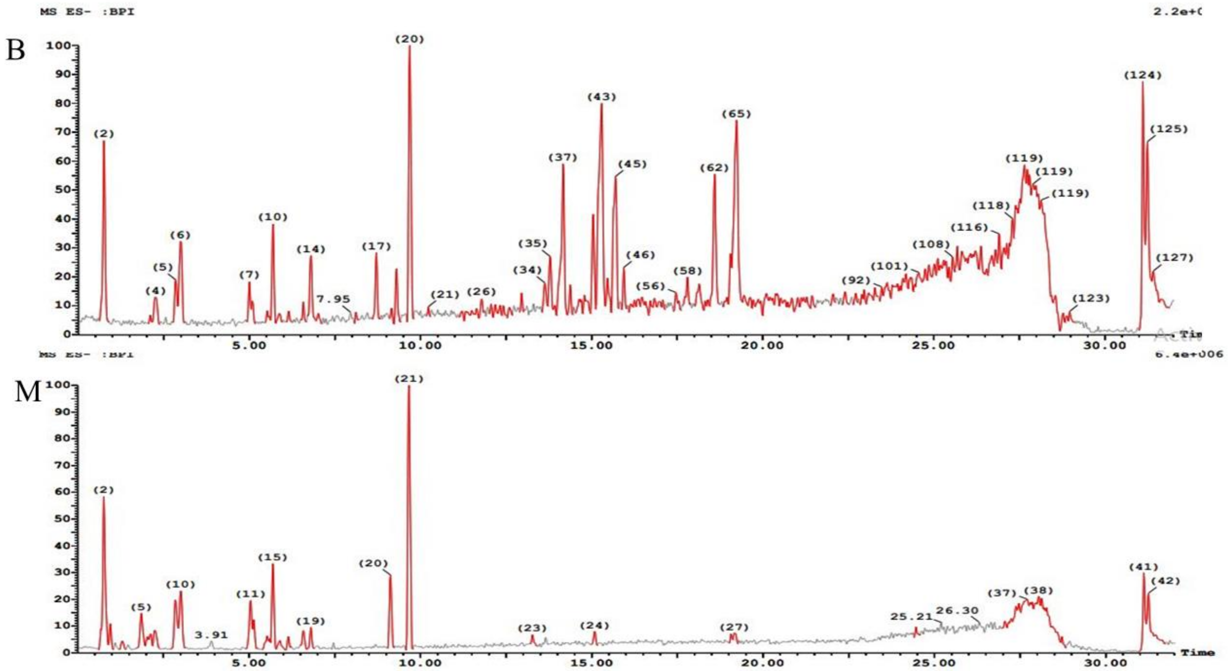

**Figure S2.** TIC of *Carissa macrocarpa* butanol (**B**), and methanol (**M**) fractions, using UPLC-ESI-MS/MS in negative ionization mode.

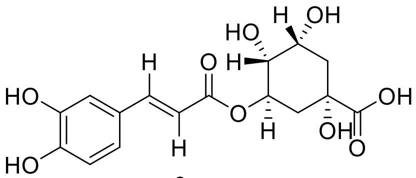
**6**

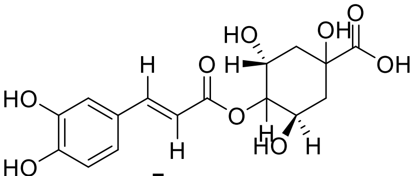
**7**

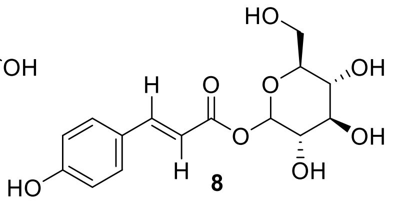
**8**

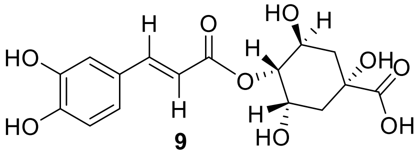
**9**

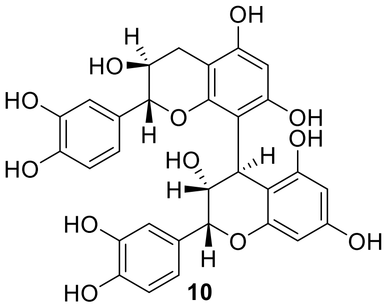
**10**

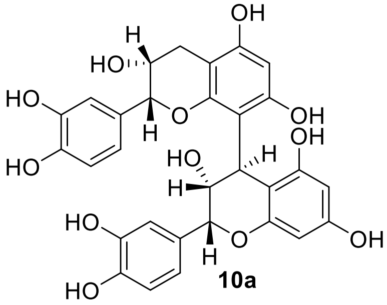
**10a**

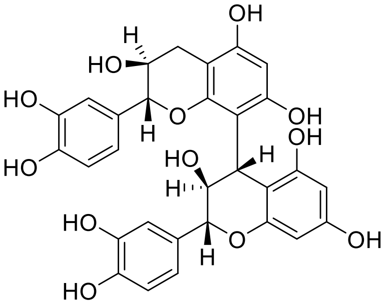
**10b**

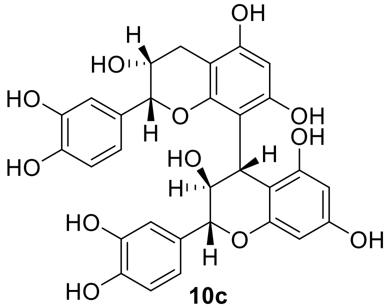
**10c**

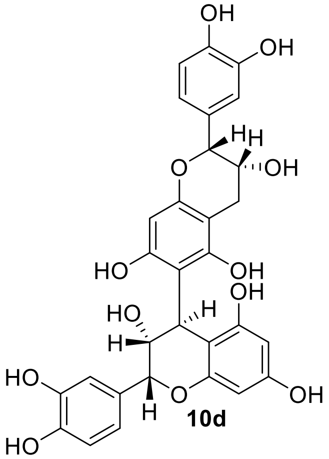
**10d**

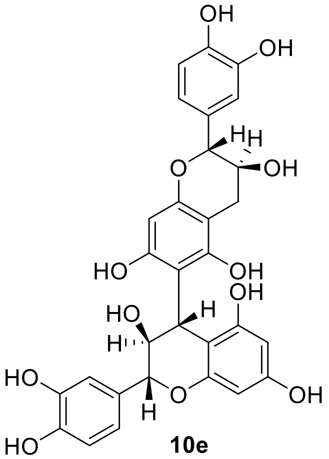
**10e**

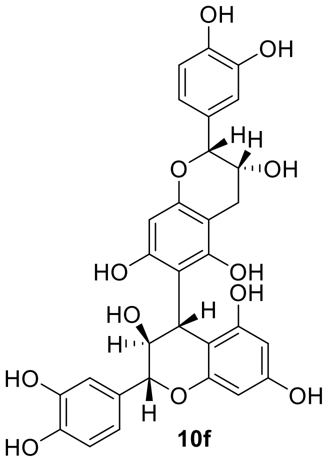
**10f**

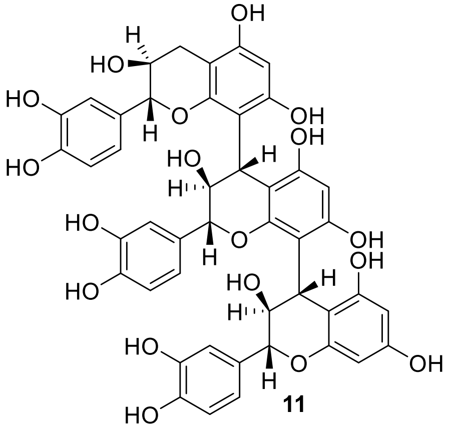
**11**

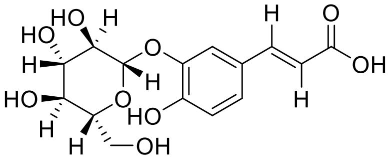
**13**

**Figure S3**. Structures of identified compounds and their isomers evaluated by molecular docking simulation.

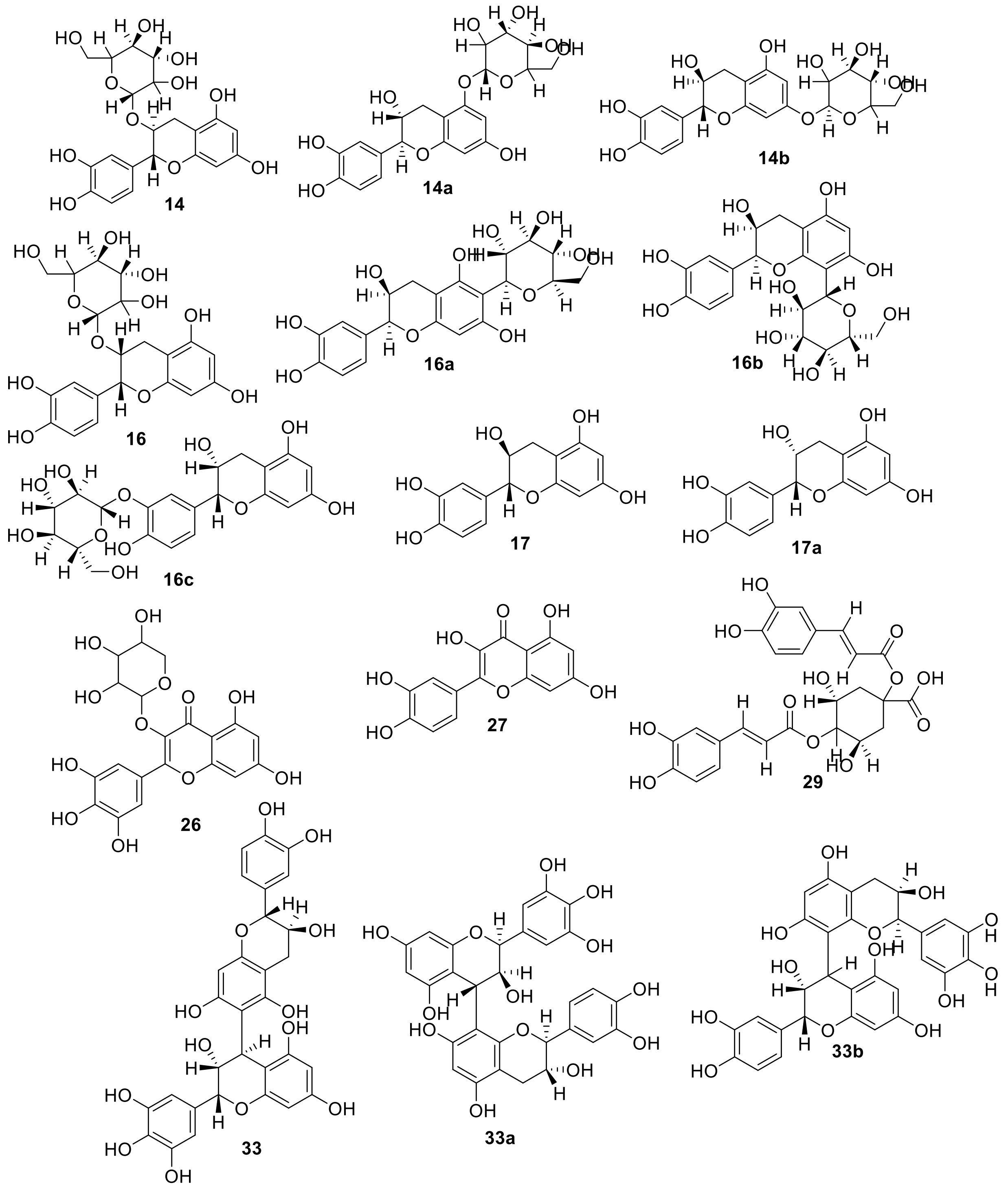


**Figure S4**. Continue structures of identified compounds and their isomers evaluated by molecular docking simulation.

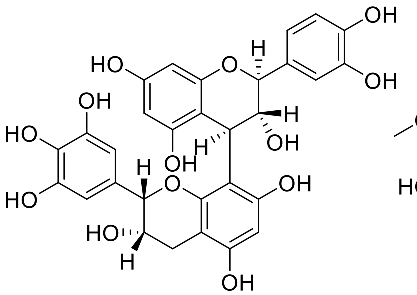

**33c**

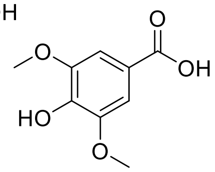

**35**

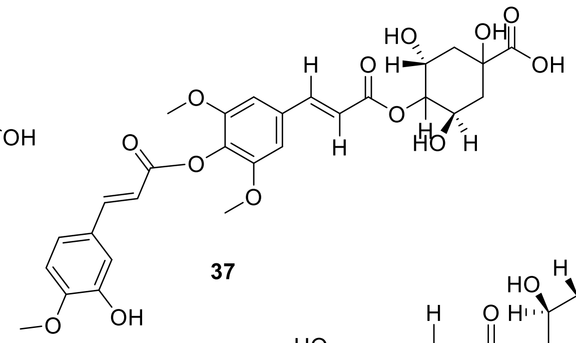

**37**

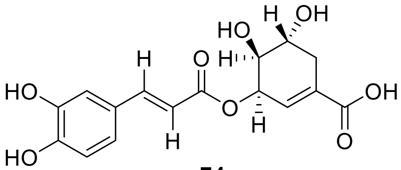

**74**

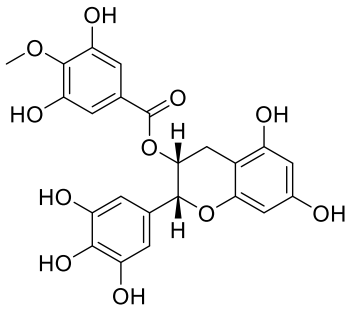

**50**

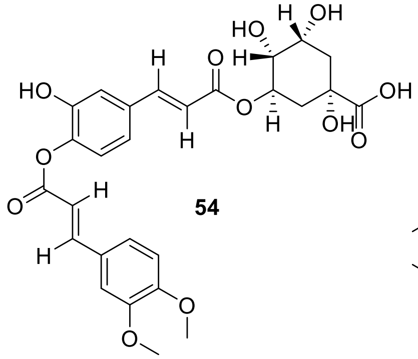

**54**

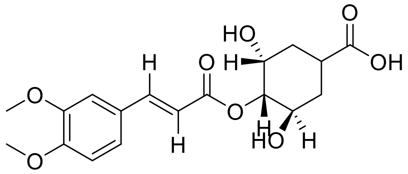

**75**

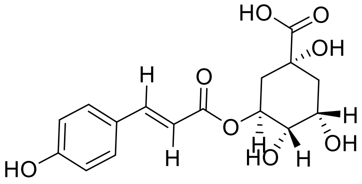

**77**

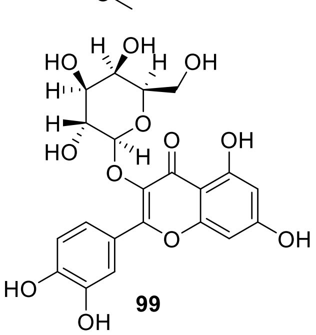

**99**

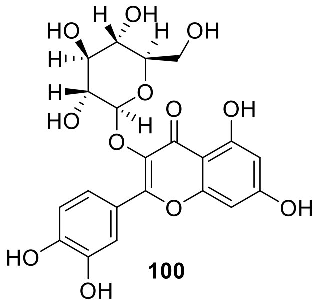

**100**

**Figure S5**. Continue structures of identified compounds and their isomers evaluated by molecular docking simulation.

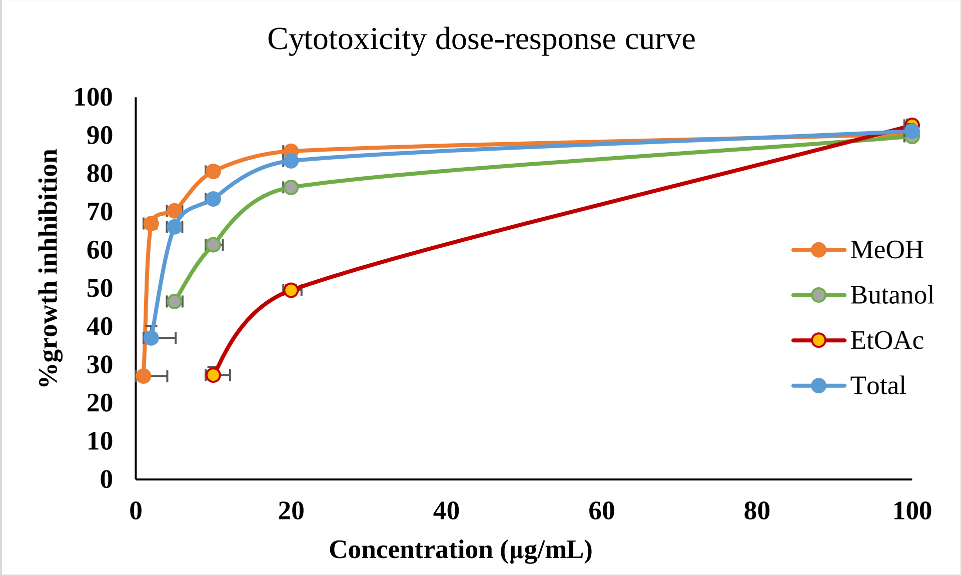


**Figure S6:** Dose-response curves of total extract and fractions of *C. macrocarpa* against A549 cell line.

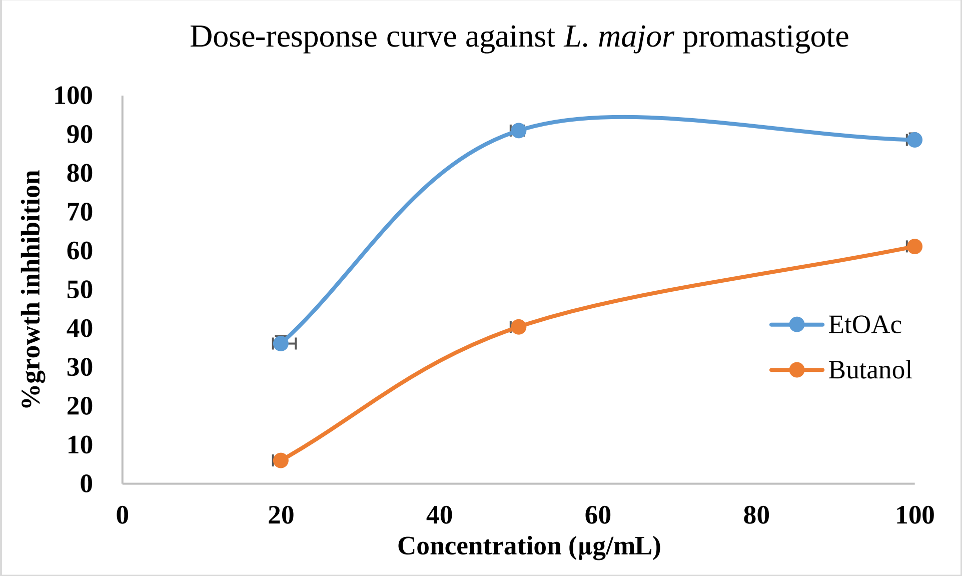


**Figure S7:** Dose-response curves of total extract and EtOAc and butanol fractions of *C. macrocarpa* against *L. major* promastigote.

## References


1. Souilem, F.; Dias, M.I.; Barros, L.; Calhelha, R.C.; Alves, M.J.; Harzallah-Skhiri, F.; Ferreira, I.C. Phenolic Profile and Bioactive Properties of *Carissa macrocarpa* (Eckl.) A. DC.: An In Vitro Comparative Study between Leaves, Stems, and Flowers. *Molecules* **2019**, *24*, 1696.
2. Ben Said, R.; Hamed, A.I.; Mahalel, U.A.; Al-Ayed, A.S.; Kowalczyk, M.; Moldoch, J.; Oleszek, W.; Stochmal, A. Tentative characterization of polyphenolic compounds in the male flowers of *Phoenix dactylifera* by liquid chromatography coupled with mass spectrometry and DFT. *Int. J. Mol. Sci.* **2017**, *18*, 512.

3. Jaiswal, R.; Matei, M.F.; Subedi, P.; Kuhnert, N. Does roasted coffee contain chlorogenic acid lactones or/and cinnamoylshikimate esters? *Food Res. Int.* **2014**, *61*, 214-227.
4. Alakolanga, A.; Siriwardane, A.; Kumar, N.S.; Jayasinghe, L.; Jaiswal, R.; Kuhnert, N. LC-MSn identification and characterization of the phenolic compounds from the fruits of *Flacourtia indica* (Burm. F.) Merr. and Flacourtia inermis Roxb. *Food Res. Int.* **2014**, *62*, 388-396.
5. Pereira, E.; Pimenta, A.I.; Calhelha, R.C.; Antonio, A.L.; Verde, S.C.; Barros, L.; Santos-Buelga, C.; Ferreira, I.C. Effects of gamma irradiation on cytotoxicity and phenolic compounds of *Thymus vulgaris L.* and Mentha x piperita L. *LWT - Food Sci. Technol.* **2016**, *71*, 370-377.
6. Keefover-Ring, K.; Ahnlund, M.; Abreu, I.N.; Jansson, S.; Moritz, T.; Albrectsen, B.R. No evidence of geographical structure of salicinoid chemotypes within *Populus tremula*. *PLoS One* **2014**, *9*, e107189.
7. Lin, L.-Z.; Chen, P.; Harnly, J.M. New phenolic components and chromatographic profiles of green and fermented teas. *J. Agric. Food Chem.* **2008**, *56*, 8130-8140.
8. Li, J.; Kuang, G.; Chen, X.; Zeng, R. Identification of chemical composition of leaves and flowers from *Paeonia rockii* by UHPLC-Q-Exactive Orbitrap HRMS. *Molecules* **2016**, *21*, 947.
9. Jaiswal, R.; Jayasinghe, L.; Kuhnert, N. Identification and characterization of proanthocyanidins of 16 members of the Rhododendron genus (Ericaceae) by tandem LC–MS. *J. Mass Spectrom.* **2012**, *47*, 502-515.
10. Patel, S. Food, pharmaceutical and industrial potential of *Carissa* genus: an overview. *Rev. Environ. Sci. Biotechnol.* **2013**, *12*, 201-208.
11. May A. El-Sayed, A.A.A.-G.; Hamdan, D.I.; El-Shazly, A.M. Phytoconstituents, LC-ESI-MS profile, antioxidant and antimicrobial activities of *Citrus* x *limon* L. Burm. f. cultivar variegated pink lemon. *J. Pharm. Sci. Res.* **2017**, *9*, 375-391.
12. Abu-Reidah, I.M.; Ali-Shtayeh, M.S.; Jamous, R.M.; Arráez-Román, D.; Segura-Carretero, A. HPLC–DAD–ESI-MS/MS screening of bioactive components from *Rhus coriaria L.* (Sumac) fruits. *Food Chem.* **2015**, *166*, 179-191.
13. Yuzuak, S.; Ballington, J.; Xie, D.-Y. HPLC-qTOF-MS/MS-based profiling of flavan-3-ols and dimeric proanthocyanidins in berries of two muscadine grape hybrids FLH 13-11 and FLH 17-66. *Metabolites* **2018**, *8*, 57.
14. Hussain, S.A.; Hameed, A.; Nazir, Y.; Naz, T.; Wu, Y.; Suleria, H.A.R.; Song, Y. Microencapsulation and Characterization of Natural Polyphenols from PHF Extract. *Nutrients* **2018**, *10*, 843.
15. Tala, V.R.S.; Candida da Silva, V.; Rodrigues, C.M.; Nkengfack, A.E.; Campaner dos Santos, L.; Vilegas, W. Characterization of proanthocyanidins from *Parkia biglobosa* (Jacq.) G. Don. (Fabaceae) by flow injection analysis—Electrospray ionization ion trap tandem mass spectrometry and liquid chromatography/electrospray ionization mass spectrometry. *Molecules* **2013**, *18*, 2803-2820.
16. Zhang, L.; Deng, W.-W.; Wan, X.-c. Advantage of LC-MS metabolomics to identify marker compounds in two types of Chinese dark tea after different post-fermentation processes. *Food Sci. Biotechnol.* **2014**, *23*, 355-360.
17. Yang, N.-Y.; Yang, Y.-F.; Li, K. Analysis of Hydroxy Fatty Acids from the Pollen of *Brassica campestris L.* var. *oleifera* DC. by UPLC-MS/MS. *J. pharmaceutics* **2013**, *2013*.
18. Moodley, R.; Koorbanally, N.; Jonnalagadda, S.B. Elemental composition and fatty acid profile of the edible fruits of Amatungula (*Carissa macrocarpa*) and impact of soil quality on chemical characteristics. *Anal. Chim. Acta* **2012**, *730*, 33-41.
19. Petta, V.; Gkiozos, I.; Strimpakos, A.; Syrigos, K. Histones and lung cancer: Are the histone deacetylases a promising therapeutic target? *Cancer Chemother. Pharmacol.* **2013**, *72*, 935-952.
20. Zhou, Y.-X.; Xin, H.-L.; Rahman, K.; Wang, S.-J.; Peng, C.; Zhang, H. Portulaca oleracea L.: a review of phytochemistry and pharmacological effects. *Biomed Res. Int.* **2015**, *2015*.
21. Kaunda, J.S.; Zhang, Y.-J. The genus *Carissa*: An ethnopharmacological, phytochemical and pharmacological review. *Nat. prod. bioprospect.* **2017**, *7*, 181-199.
22. Jiao, Q.-S.; Xu, L.-L.; Zhang, J.-Y.; Wang, Z.-J.; Jiang, Y.-Y.; Liu, B. Rapid characterization and identification of non-diterpenoid constituents in *Tinospora sinensis* by HPLC-LTQ-orbitrap MSn. *Molecules* **2018**, *23*, 274.
23. Yu, J.-Q.; Xian, Y.-X.; Geng, Y.-L.; Wang, D.-J.; Zhou, H.-L.; Wang, X. Anti-liver cancer constituents from the thorns of *Gleditsia sinensis*. *Phytochem. Lett.* **2015**, *13*, 343-347.
24. Izhar, S.; Ahmed, D. *Carissa opaca*: A plant with great potential for future drugs for degenerative and infectious diseases. *ChemistrySelect* **2016**, *1*, 3005-3011.
